# Psychological Stress as a Modulator of Functional Recovery Following Spinal Cord Injury

**DOI:** 10.3389/fneur.2014.00044

**Published:** 2014-04-09

**Authors:** Sioui Maldonado Bouchard, Michelle A. Hook

**Affiliations:** ^1^Department of Neuroscience and Experimental Therapeutics, Texas A&M Health Science Center, Texas A&M Institute for Neuroscience, College of Medicine, College Station, TX, USA

**Keywords:** psychological stress, glucocorticoids, inflammation, glucocorticoid resistance, spinal cord injury, NF-κB, Bcl-2, apoptosis

## Abstract

There is strong evidence indicating that the social environment triggers changes to the psychological stress response and glucocorticoid receptor function. Considerable literature links the subsequent changes in stress resiliency to physical health. Here, converging evidence for the modulatory role of chronic psychological stress in the recovery process following spinal cord injury (SCI) is presented. Despite the considerable advances in SCI research, we are still unable to identify the causes of variability in patients’ recovery following injury. We propose that individuals’ past and present life experiences (in the form of stress exposure) may significantly modulate patients’ outcome post-SCI. We propose a theoretical model to explain the negative impact of chronic psychological stress on physical and psychological recovery. The stress experienced in life prior to SCI and also as a result of the traumatic injury, could compromise glucocorticoid receptor sensitivity and function, and contribute to high levels of inflammation and apoptosis post-SCI, decreasing the tissue remaining at the injury site and undermining recovery of function. Both stress-induced glucocorticoid resistance and stress-induced epigenetic changes to the glucocorticoid receptor can modulate the nuclear factor-kappa B regulated inflammatory pathways and the Bcl-2 regulated apoptosis pathways. This model not only contributes to the theoretical understanding of the recovery process following injury, but also provides concrete testable hypotheses for future studies.

In 2001, at the 54th World Health Assembly, the 191 Member States of the World Health Organization approved the International Classification of Functioning, Disability, and Health. This classification called for a shift in the way medicine views health and disability. It recognized that an injury occurs in a social context, and thus, that not only physical signs but also environmental elements should be taken into consideration by doctors and medical researchers in the quest to better predict and improve patients’ outcome post-injury (1). Despite this initiative, more than a decade later, outcome prediction following a spinal cord injury (SCI) remains predominantly based on injury-related factors alone, and its accuracy is limited at best (2). We posit that psychological stress inherent to the present and past environments of SCI patients may account, in part, for the unexplained variance in functional recovery.

The link between a stressful environment and disease recovery is well recognized in diverse clinical fields (3). Psychological stress is associated with greater risk of asthma exacerbation (4), increases in cardiovascular disease (5), and faster progression of HIV/AIDS (6). However, despite the fact that decreased psychological well-being post-SCI is strongly documented clinically, understanding of its role in recovery is lacking. As many as 60% of patients suffer from depression (7), anxiety (8), and general decreased quality of life (9). Many patients also experience loneliness; the perception of few people who understand them and in whom they can confide (10). Interestingly, however, not only does the psychological health of SCI patients improve in a positive social environment, but *physical* health is also benefited. Conducting a systematic review of the literature on patients with SCI, Muller et al. (11) found a positive correlation between social support and physical health, mental health, and pain management post-injury. Patients with a strong social support system showed better health and functioning (11). In 2011, a multilevel modeling, retrospective analysis of cross-sectional survey data also indicated that individual socioeconomic status strongly predicts outcome post-SCI (12). Saunders et al. (13) further found household income to be inversely correlated with pressure ulcers in patients with SCI, even after controlling for demographic and injury variables. Finally, a number of epidemiological studies have found that patients with good coping skills have a more positive experience after SCI, and display a lower incidence of depression (14). These analyses highlight the potential impact of a positive environment, and by extension a stressful environment, on the recovery of patients following SCI.

Both pre and post-SCI environment may influence recovery. Indeed, both a past and present stressful environment, including the traumatic experience of SCI, can alter the function of the glucocorticoid receptor, which is involved in both the stress response and the activity of protein complexes involved in inflammation and apoptosis, processes critical in SCI injury and recovery (Figure 1). Chronic psychological stress can compromise glucocorticoid receptor sensitivity and function (15–17), and this may contribute to high levels of inflammation and apoptosis, decreasing the tissue remaining at the injury site and undermining recovery of function post-SCI. What is more, who is most susceptible to stress as a result of the traumatic injury may depend on the stress experienced in life prior to SCI. While numerous other receptors and proteins involved in SCI and recovery processes (i.e., excitotoxicity, oxidative stress, and glial activation) could be affected by a chronically stressful environment, this review focuses on the impact of glucocorticoid receptors. There is strong evidence for the social environment triggering changes to the psychological stress response, and glucocorticoid receptor function, with considerable literature linking the subsequent changes in stress resiliency to physical health.

**Figure 1:**
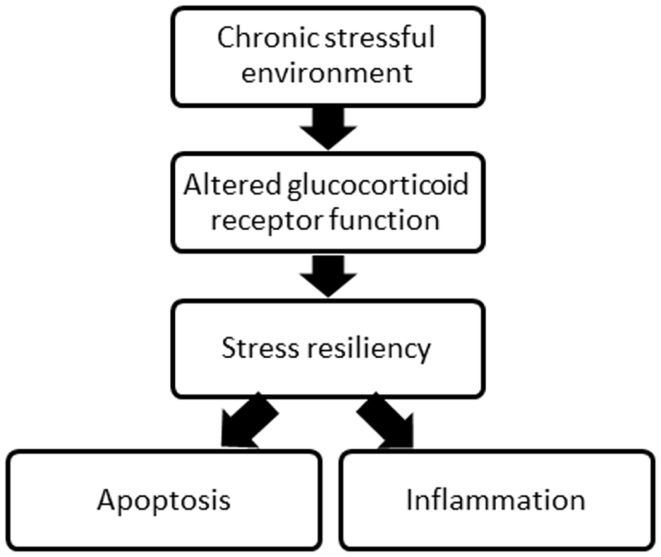
**A schematic of the overall concept proposed relating the environment, glucocorticoid receptor function, stress resiliency, and two major processes following SCI**.

## Stress Resiliency Modulates Inflammation and Apoptosis

Psychological stress is defined as an “emotional experience accompanied by predictable biochemical, physiological, and behavioral changes” (18). Examples include experiences of social isolation, rape, verbal and physical abuse, traumatic injury, or war. A homeostatic challenge triggers a stress response, via both a fast route (neural), involving the autonomic nervous system (often referred to as the fight-or-flight response), and a slower route (endocrine), involving the hypothalamic–pituitary–adrenal axis. When we are exposed to a homeostatic challenge, such as a sign of danger, the brain first reacts via the neural route. It activates the sympathetic nervous system, which prompts the adrenal glands to release catecholamines, namely epinephrine and norepinephrine. Also activated more slowly is the hypothalamic–pituitary–adrenal axis, the endocrine route. An outside challenge will lead the hypothalamus to secrete corticotropin-releasing hormone (CRH) into the pituitary portal circulation. This, in turn, stimulates the pituitary glands to release the adrenocorticotropic hormone (ACTH), which triggers glucocorticoid release by the adrenal glands (19). Glucocorticoids function in a negative neuroendocrine feedback loop; high plasma levels signal the hypothalamus to stop producing CRH, and signal the anterior pituitary to stop producing ACTH. The effects of stress on glucocorticoid receptor signaling and the downstream processes of inflammation and apoptosis, however, depend on whether such stress is acute or chronic.

### Acute stress response

In response to acute stress, glucocorticoids and their receptors enhance cellular resiliency and plasticity through inhibition of inflammation and apoptosis. Glucocorticoids are found in the bloodstream, where 90% of these molecules are bound to glucocorticoid-binding globulin. Only unbound glucocorticoids, the remaining 10%, can cross the blood–brain barrier and cell membranes (19). The molecules that do cross the membrane of cells, and reach the cytoplasm, bind to two types of receptors: mineralocorticoid receptors and glucocorticoid receptors. Mineralocorticoid receptors have an affinity to glucocorticoids 10 times greater than that of glucocorticoid receptors (20). Thus, of the percentage of glucocorticoid molecules that are able to cross cell membranes, only a fraction actually bind to glucocorticoid receptors (19).

**Figure 2:**
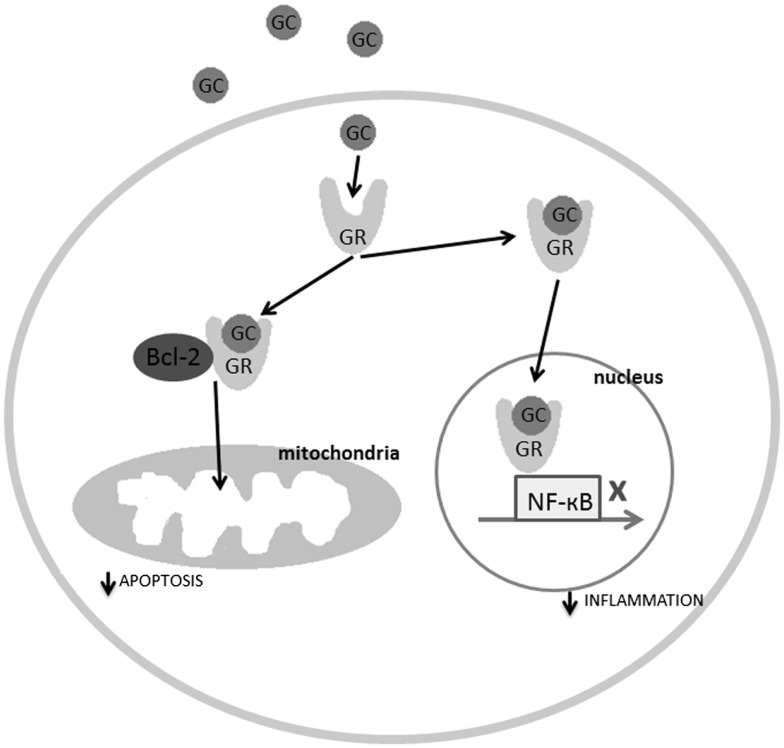
**The molecular mechanisms explaining the regulatory role of the ligand-bound glucocorticoid receptors in inflammation and apoptosis**. GC, glucocorticoids; GR, glucocorticoid receptor.

Glucocorticoid receptors are found in virtually all cells of the body, including, importantly, in the microglia of the spinal cord (21, 22). In normal circumstances, glucocorticoids are anti-inflammatory. In the cytoplasm, they can bind to glucocorticoid receptors, and trigger conformational changes, which allow the glucocorticoid receptors to translocate to the cell nucleus via microtubular highways (23). Once in the nucleus, the glucocorticoid receptors can act as ligand-binding transcription factors (24). Nuclear glucocorticoid receptors regulate transcription in conjunction with a number of transcription factors, among them the activator protein 1 (AP-1) and nuclear factor-kappa B (NF-κB), which are major initiators of inflammation (see Figure 2). In the spinal cord, ligand-bound glucocorticoid receptors inhibit the activity of NF-κB, thereby impeding immune cells from expressing certain pro-inflammatory cytokines such as interleukin-6 (IL-6), tumor necrosis factor alpha (TNF-α), or IL-1β (25, 26). This occurs in microglia, but also neurons, as they can both express NF-κB (27). The inverse is also true; transcription factors involved in inflammation, such as NF-κB, can regulate the expression of the glucocorticoid receptor gene, by repressing certain promoters of this gene (26). This is referred to as reciprocal repression.

Glucocorticoid receptors, in addition to being involved in reciprocal repression with inflammation-related transcription factors, regulate the activity and expression of kinases and phosphatases such as mitogen-activated protein kinases (MAPKs), cyclin-dependent kinases (Cdks), dual-specificity phosphatases (DUSPs), and protein Y phosphatases (26). Kinases and phosphatases are essential in inflammation. They transfer and remove phosphate groups from specific substrates involved in inflammation signaling pathways, and play an important role in initiating the expression of pro-inflammatory genes. For example, in order for the IKK-NF-κB (inhibitors of kappa B kinase-NF-κB) pathway to be activated, a pathway which triggers tissue inflammation, the inhibitor of NF-κB must be dissociated (28). This is done by NF-κB-specific kinases which phosphorylate this inhibitor, thus freeing NF-κB from inhibition (26). Glucocorticoid receptors inhibit inflammatory signals by inhibiting the activity of transcription factors that initiate the expression of pro-inflammatory cytokine genes, and by inhibiting the expression of kinases and phosphatases that initiate inflammation signaling pathways.

In addition to anti-inflammatory effects, glucocorticoid receptors also possess anti-apoptotic properties. It is well-known and regular medical practice, to administer one high dose of prednisone (a corticosteroid) to patients who are extremely ill and at-risk of dying, as in the case of severe SCI (29, 30). This treatment has a dramatic effect on the patient, stabilizing his condition within minutes. This is partly because glucocorticoid receptors not only travel to the cell nucleus to inhibit pro-inflammatory cytokine gene expression, but also travel to the mitochondria to inhibit apoptosis. Specifically, ligand-bound glucocorticoid receptors in the cytoplasm can bind to B-cell CLL/lymphoma 2 (Bcl-2), which is an anti-apoptotic regulator inhibited by the gene *Bad* post-SCI. The ligand-bound glucocorticoid receptor-Bcl-2 complex travels to the mitochondria and halts the apoptosis cascade consisting of cytochrome *c* release and caspase activation (31) (see Figure 2). Studies have also shown that low doses of glucocorticoids increase mitochondrial oxidation (necessary for ATP synthesis), mitochondrial membrane potential, and mitochondrial calcium holding capacity (32). In short, glucocorticoid receptors inhibit inflammation and apoptosis in response to acute stress.

This modulatory role of glucocorticoids, for inflammation and apoptosis, has been utilized in the clinical treatment of SCI. Methylprednisolone remains the only option for therapeutic intervention in the emergency management of SCI (33), despite conflicting results both in laboratory experiments and clinical trials (34–36). Indeed, some researchers have questioned clinical trial results (37–39), while others have criticized such evaluations for focusing solely on statistical details and theoretical safety concerns (33, 40). A major concern is that the side effects of methylprednisolone outweigh its modest functional benefits. Dose curve and timing studies may be necessary to evaluate other possibly beneficial treatment regimens, such as a low methylprednisolone dose administration over an extended time period. At present, however, the clinical use of methylprednisolone remains controversial, and while a full discussion of this controversy is beyond the scope of this review [for both sides of the issue, the reader is referred to Ref. (33, 40)] empirical data does support the idea that glucocorticoids reduce apoptosis (41).

Indeed, early animal studies on the use of glucocorticoids following SCI posited that methylprednisolone improved recovery by reducing secondary processes of injury, such as secondary ischemia (42), lipid peroxidation, (42, 43) and apoptosis (44). Because the methylprednisolone dose demonstrating some success in the multicenter, double-blind randomized clinical trial NASCIS II was much higher (30 mg/kg bolus, 5.4 mg/kg maintenance per hour) than that required for glucocorticoid receptor activation, it has also been suggested that the beneficial properties of methylprednisolone treatment following SCI may not involve the glucocorticoid receptors, or at least not only involve glucocorticoid receptor related mechanisms (30). Bracken et al. (30) suggested that instead, methylprednisolone at such a high dose may work through its facilitation of blood flow through the injured cord and inhibition of lipid peroxidation. In any case, based on the modulation of inflammation and apoptosis discussed above, there should be potential for the use of glucocorticoids from a theoretical perspective.

The timing of a therapeutic intervention, in regulating the glucocorticoid response, would be critical for efficacy. Interestingly, the efficacy of methylprednisolone is also contingent on the timing of the therapeutic intervention (30, 34). Various pro-inflammatory genes are up-regulated immediately following SCI. For example, the NF-κB gene, which acts as a transcription factor to initiate the transcription of various gene types, including genes encoding for pro-inflammatory cytokines such as IL-6 and TNF-α (45, 46), is up-regulated as early as 30 min post-injury in a rodent model of contusion injury (47). This up-regulation continues for at least 72 h, and occurs in neurons, macrophages (microglia), and endothelial cells of the injured spinal cord (47). Methylprednisolone, which is generally considered to have the greatest therapeutic impact when administered in the 8-h following SCI (30), has been found to inhibit the activation of NF-κB and downstream production of pro-inflammatory cytokines such as TNF-α (48). Indeed, in one early rodent study, methylprednisolone administered at 30 mg/kg intravenously inhibited IL-6 expression and NF-κB activity by 55% (48).

Recently, it has been suggested that inhibiting the inhibitor of κB kinase (IKK) might also be useful for post-SCI therapy. IKK, by phosphorylating the IkB, leads to its ubiquitination and degradation by proteases, thus activating the NF-κB complex (49). In a rodent study of SCI, inhibiting IKK was found to prevent neutrophil infiltration as well as inhibiting the activity of caspase-3 (by modulating Bcl-2 expression), thus reducing apoptosis in the injured spinal cord (50). These studies support the potential of methylprednisolone treatment, modulating NF-κB and Bcl-2 activity in the acute phase of SCI. However, methylprednisolone is not always an efficient line of treatment (36). The past and present environment experienced by the patient may critically modulate the molecular cascades activated with glucocorticoid administration. Indeed, exposure to a chronic stressor is linked to glucocorticoid resistance and epigenetic changes that may result in increased unbound glucocorticoid expression and exacerbation of inflammation and apoptosis.

### Chronic stress: Glucocorticoid resistance and epigenetic modifications

#### Glucocorticoid resistance

While the acute stress response has been linked to reduced inflammation, chronic psychological stress can result in systemic inflammation (15, 51) and the development of glucocorticoid resistance. Chronic stress-induced inflammation has been suggested to play a central role in autoimmune diseases (52), infectious diseases (51, 53), and cardiovascular conditions (54), among others. Glucocorticoid receptors play an integral role in the transition from the protective effects of acute stress to the negative consequences of chronic stress. Chronic stress causes extended activation of the hypothalamic–pituitary–adrenal axis and the autonomic nervous system. This extended exposure to glucocorticoid (55), epinephrine, and norepinephrine hormones (56) results in a diminished expression and function of the glucocorticoid receptor (15). Indeed, chronic exposure to elevated levels of glucocorticoids due to protracted steroid treatment (57), chronic inflammation (58, 59), early-life trauma (60, 61), or chronic psychological stress (15, 51), can lead to the development of glucocorticoid resistance: a decreased glucocorticoid receptor sensitivity. As a result of glucocorticoid resistance, immune cells lose sensitivity to glucocorticoids, the hormone which usually serves to put a stop to inflammatory responses (62).

Glucocorticoid resistance due to chronic psychological stress not only hinders the inhibition of pro-inflammatory gene expression in the cell nucleus by impeding ligand-bound glucocorticoid receptors from inhibiting NF-κB activity, but can also impair the anti-apoptotic role of glucocorticoid receptors in the mitochondria. Recent *in vitro* and *in vivo* studies have indicated that whereas low doses or one single high dose of glucocorticoids enable the anti-apoptotic effects of the glucocorticoid receptors, chronically high glucocorticoid levels, due to chronic stress, lead to decreased levels of glucocorticoid receptors and anti-apoptotic protein chaperone Bcl-2 in the mitochondria (32, 63). These mechanisms are of high relevance to SCI patients, as chronic inflammation and apoptosis contribute to neural loss, motor impairment (64), the development of chronic pain (65, 66), anxiety (67), and depression (68, 69).

Although the exact mechanism through which chronic stress leads to glucocorticoid resistance is not fully understood, studies of glucocorticoid resistant animal species point to the role of FK506-binding protein 51 (FKBP51) overexpression and heat shock protein 90 (70). FKBP51 is an immunophilin protein that is part of the heterocomplex of the mature glucocorticoid receptor, and regulates its sensitivity. The *FKBP5* gene, which encodes for FKBP51, is up-regulated in response to chronic psychological stress or psychological trauma (71). Recent studies have uncovered an association between *FKBP5* dysregulation and individuals’ attention to threats as a result of racial discrimination (71). It also seems that *FKBP5* dysregulation could play a role in the vulnerability to post-traumatic stress disorder in war veterans (72) and stress-related psychiatric illness in individuals having experienced childhood trauma (73). Specifically, the ligand-bound glucocorticoid receptors have been shown to up-regulate the expression of FKBP51 (74, 75). When the FKBP51 chaperone interacts with heat shock protein 90, it lowers the affinity of the glucocorticoid receptor heterocomplex to glucocorticoids (76). In other words, chronic psychological stress increases levels of glucocorticoids; ligand-bound glucocorticoid receptors increase levels of FKBP51, and FKBP51 (bound to heat shock protein 90) in turn decreases the affinity of the glucocorticoid receptor to glucocorticoids, further increasing levels of unbound glucocorticoids. Notably in SCI, the expression of heat shock protein 90 is increased (77).

#### Epigenetic changes

A stressful environment can affect the sensitivity of the glucocorticoid receptors (51), but, critically, it can also alter its expression (60, 61, 78) via epigenetic mechanisms. Epigenetics is the study of changes in gene expression that can be triggered by environmental factors and which do not result in alterations to the DNA itself (79, 80). Environmental factors, such as diet, infections, and drugs, but also present and past psychosocial environmental factors, such as social support, psychotherapy, loneliness, as well as exposure to physical and mental abuse, may trigger epigenetic changes in SCI patients which either promote, or impede their recovery. In a pioneering study, Meaney and colleagues demonstrated that high levels of early-life licking and grooming behavior from a fostering rat mother leads to a specific tableau of stress resilience in the pups as adults (61). They showed that a positive social environment increased expression of the glucocorticoid receptor in the hippocampus, strengthened glucocorticoid feedback sensitivity, reduced the expression of the hypothalamic corticotrophin-releasing hormone, and culminated in a milder hypothalamic–pituitary–adrenal stress response (61, 81–83). At the molecular level, a rich early-life social environment (high maternal care) increases levels of 5-HT, and through a 5-HT signaling cascade, leads to high levels of nerve growth factor-inducible protein A (NGFI-A) (82). Rodent studies have shown that NGFI-A is a transcription factor that binds to the promoter of the glucocorticoid receptor gene NR3C1 at exon 17. NGFI-A recruits histone acetyltransferases (i.e., CREB-binding protein) and together they enable the demethylation of the glucocorticoid receptor promoter region (81). By acetylating the promoter region, and demethylating it, a rich early-life environment thus triggers an epigenetic change at the promoter region of the glucocorticoid receptor gene, which results in its up-regulation. The resulting increase in glucocorticoid receptor concentration in turn translates into a healthier stress response (less circulating glucocorticoids following stress) (84).

Critically, the reverse is also true. A stressful early-life environment such as that caused by poverty, abuse, rape, or war can induce powerful epigenetic changes. For example, epigenetic modifications to the glucocorticoid receptor have been found in individuals that have suffered childhood abuse, and these modifications dramatically increased their risk of suicide (60). Stressful environments lead to a decrease in glucocorticoid receptor expression, since the signaling cascade described above cannot be initiated, and the promoter of the glucocorticoid receptor gene is therefore not demethylated (81, 82). This epigenetic alteration remains until adulthood. The resulting down-regulation of the glucocorticoid receptor leads to chronically increased levels of circulating glucocorticoids, reduced glucocorticoid feedback sensitivity, and increased anxiety among other effects (61, 84, 85). The implications of this tableau are higher susceptibility to stress-related health problems, such as autoimmune diseases, cardiovascular diseases, anxiety, and depression – all observed following SCI.

Epigenetics is already used in the development of anti-cancer therapeutic drugs in oncology (86), and neuropsychiatry (87, 88). Proposals have also been presented highlighting the potential of epigenetics in controlling chronic pain (89–91). Even in injury recovery research, scientists have begun to recognize the potential of epigenetics, namely, in traumatic brain injury and stroke (92–94). Yet, little is currently known about the role of environmentally triggered epigenetic changes in SCI and recovery.

### Chronic stress and SCI

Glucocorticoid resistance and epigenetics explain two different modifications of glucocorticoid receptor function, but one can safely argue that both may occur in synergy. When individuals suffer an SCI, for example, placing them at high-risk of suffering from chronic psychological stress (95, 96), past exposure to a stressful environment resulting in epigenetic changes that compromise glucocorticoid receptor sensitivity and function would lead to high levels of inflammation and apoptosis post-SCI, decreasing the tissue remaining at the injury site and undermining recovery of function.

**Figure 3:**
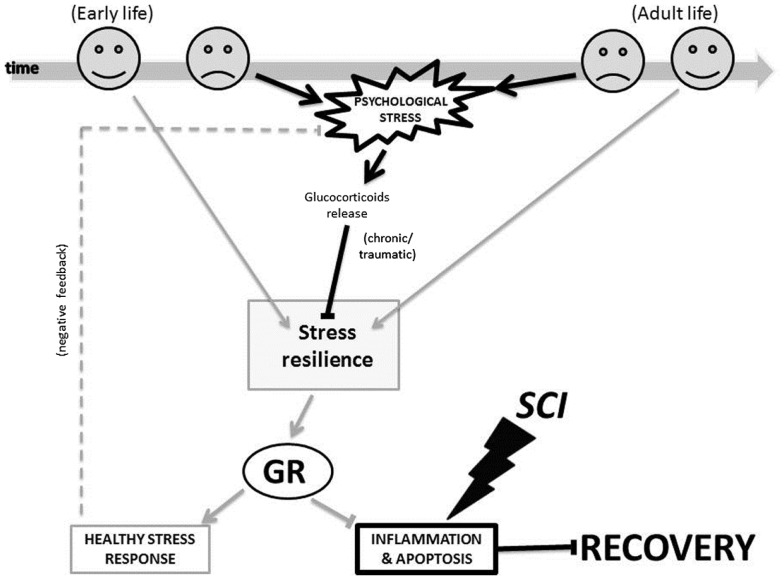
**The glucocorticoid receptor mechanism proposed for how environment-triggered epigenetic changes affect SCI recovery**. The bold black lines indicate a negative process and the light gray lines indicate a positive process in our model. Arrows (→) indicate that a process is enabled, and bars (–|) indicate that a process is inhibited. The dotted line indicates a feedback loop. GR, glucocorticoid receptor; SCI, spinal cord injury.

Figure 3 illustrates the proposed glucocorticoid receptor mechanism through which changes triggered by the early-life and adult life environment could affect patients’ psychological stress response, and it in turn could influence processes of inflammation and apoptosis important to SCI injury and recovery. In early-life, a non-stressful environment (indicated by the happy face icon in Figure 3), can promote stress resilience. This in turn leads to decreased levels of circulating glucocorticoids, and a decreased psychological stress response. Conversely, a stressful environment (indicated by the sad face icon in Figure 3) caused, for example, by physical or emotional abuse, poverty, or racism can decrease stress resilience. As a result, there are increased levels of circulating glucocorticoids, and an increased psychological stress response. Likewise, following SCI in adult life, a “non-stressful” environment (i.e., strong social support and good coping skills) may contribute to decreased psychological stress response. Again, in this case too, a stressful environment (for example due to lack of social support, perceived loneliness, or discrimination) may contribute to an increased psychological stress response. Importantly, the glucocorticoid receptors are not only involved in the stress response, but also in the inflammation and apoptosis processes. Therefore, as shown in Figure 3, these mechanisms do not only impact the psychological stress response, but also the processes of inflammation and apoptosis.

In addition to the increase in inflammation due to chronically elevated glucocorticoids levels and the glucocorticoid resistance that ensues, protracted exposure to cytokines, as a result of illness (i.e., SCI) or chronic stress, can also affect glucocorticoid receptor function and lead to increased inflammation. The signaling pathways of pro-inflammatory cytokines, such as NF-κB, MAPKs, and cyclooxygenase can affect the translocation of glucocorticoid receptors from the cytoplasm of the cell to the nucleus, or the ability of the glucocorticoid receptor to function as a transcription factor (97, 98). The result is increased production of pro-inflammatory cytokines.

This is paramount to SCI research, as chronic elevated pro-inflammatory cytokine levels can harm functional recovery, and contribute to the development of chronic pain (64, 99). Indeed, chronic inflammation resulting from the injury leads to a wide range of clinical conditions such as decreased pulmonary function (100), neuropathic pain (101–103), compromised physical recovery (104, 105), and exacerbated depressive symptoms (106). Moreover, SCI-induced chronic inflammation can result in infections and cardiovascular diseases, two leading causes of death after SCI (107).

## Clinical Significance

We have illustrated one mechanism through which environmentally triggered changes in stress resilience can affect the health of patients with SCI. We have reviewed how the environment can affect the function of the glucocorticoid receptor, which in turn will affect anti-inflammatory and anti-apoptotic cellular processes in the injured spinal cord. When the glucocorticoid receptors’ function is compromised by environment-triggered changes in stress resiliency, the protective effects of glucocorticoids is diminished, resulting in increased inflammation and apoptosis.

Critically, these mechanisms involving both pre- and post-SCI environments may work in synergy to influence the function of the glucocorticoid receptor. It can be predicted, for example, that a stressful pre-SCI environment combined with a stressful post-SCI environment may both increase the psychological stress response, triggering changes in stress resilience, which will result in decreased glucocorticoid receptor function. Glucocorticoid receptors’ function to inhibit inflammation and apoptosis by inhibiting NF-κB and aiding Bcl-2, respectively, will therefore be compromised. This may increase inflammation and apoptosis, both processes harmful to SCI recovery, as they can contribute to chronic pain, depression, neural loss, and motor impairment. In sum, given the modulatory role of the glucocorticoid receptor in the NF-κB and Bcl-2 pathways, patients’ environment and their psychological stress response are likely to play a role in their recovery after SCI. Indeed, these factors may partially explain individual differences in functional gain.

Given the modulatory role of the glucocorticoid receptor in the NF-κB and Bcl-2 pathways of inflammation and apoptosis, respectively, the environment-triggered epigenetic activation of the glucocorticoid receptor gene by acetylation and demethylation may also hold the potential to reduce inflammation and apoptosis post-SCI. The potential of this approach is underscored by findings of neuroprotective properties of valproic acid after SCI. Valproic acid up-regulates the expression of the anti-apoptotic regulator Bcl-2 (108, 109). By inhibiting deacetylation, it increases the expression of the brain-derived neurotrophic factor (BDNF) and glial cell-derived neurotrophic factor (GDNF), among others, thus helping regenerate damaged neurons (110). Abdanipour et al. (109) recently found that intraperitoneal administration of valproic acid to spinally contused rats reduced mRNA expression of cytokines that regulate inflammation. Administration of this HDACi thus helped to reduce secondary damage. Subjects who received a dose of 400 mg/kg showed higher locomotor recovery scores (Basso, Beattie, and Bresnahan rating scale) than non-treated contused subjects by day 28 post-injury (109). Research on the epigenetic changes induced by a stressful environment could help develop psychosocial therapies targeted to at-risk patients so that they may cope better with the stress post-SCI and consequently have a more successful recovery.

Indeed, a combination of epigenetic-based pharmacotherapy with psychosocial therapy may be optimal given their possible synergistic effects. In a mouse traumatic brain injury model, an enriched environment post-injury was recently shown to improve memory function. The enriched environment, consisting of housing four mice together in a cage with a running wheel and toys, increased general acetylation and methylation of histone 3 and 4 in the hippocampus and cortex, thus facilitating synaptic plasticity (111). Based on this work, Dash et al. (112) then showed that behavioral training also improves spatial learning and memory post-traumatic brain injury. Furthermore, they found that an HDACi, sodium butyrate, potentiated the level of improvements observed in traumatic brain injured mice, when, and only when, combined with concurrent behavioral training. In other words, histone deacetylase inhibitor treatment alone did not improve memory post-traumatic brain injury, and receiving behavioral training prior to HDACi treatment did not improve outcome either; it is the combination of both behavioral therapy and pharmacotherapy that promoted recovery. Given that the spinal cord is capable of learning (113, 114), it is possible that SCI recovery too may benefit from HDACi treatment administered concurrent with physical training.

Overall, these epigenetic and psychological stress findings underscore the importance of including psychological evaluations and treatments in the post-SCI intervention. Greater collaboration between the various types of health care providers involved in the post-SCI treatment plan, including physicians, psychiatrists, nurses, psychologists, physical therapists, occupational therapists, and speech therapists, will be essential in providing more successful treatment. For example, if a psychologist and psychiatrist, with the help of the patient and his family, can determine whether the patient has experienced traumatic events prior to the injury, or has any history of anxiety or depression, a treatment plan including more immediate and elaborate psychological therapy could be provided to minimize the negative consequences of SCI-associated stress. Likewise, nurses could be informed to encourage a social support team for the patient, composed of family members, friends, and even hospital volunteers, in order to promote the patient’s psychological well-being. These seemingly small changes, in addition to formal psychosocial therapy and drug treatment could be favorable to the patients’ recovery.

## Conclusion

We have reviewed one mechanism, via the function of the glucocorticoid receptor and the psychological stress response to the environment, which may account for a part of the unexplained variance found in patients’ recovery following SCI. Based on the current literature, two potential mechanisms involved are the processes of inflammation and apoptosis: the NF-κB and the Bcl-2 pathway, respectively. It must be noted that the action of glucocorticoid receptors is only one pathway that may explain the effect of the environment on spinal cord. Certainly, other relevant pathways involve the reaction of immune cells such as lymphocytes, macrophages, monocytes, natural killer cells, and T cells to the glucocorticoids and catecholamines released by the brain following a stressor. For instance, glucocorticoids and catecholamines inhibit the expression of Substance P (115), a neuropeptide, and its receptor (116). This neuropeptide induces macrophages to release pro-inflammatory cytokines, such as IL-1, IL-6, and TNF-α, as well as prostaglandin and thromboxane, also involved in inflammation (117). Likewise, in the central nervous system, stress-triggered release of glucocorticoids activates microglia, and sensitizes them to future inflammatory insults (118). The potential synergistic effects of these various glucocorticoid–immune interactions are likely to attenuate physical recovery after SCI.

Regulation of these substrates through epigenetic mechanisms may significantly improve SCI recovery, not only by opening a window of opportunities for new targeted and personalized treatments combining both psychosocial therapies and epigenetic therapies, but also by allowing physicians to identify SCI patients at higher risk for a more difficult recovery – both physical and psychological. Future research will be essential in determining the main epigenetic modifications that positively or negatively affect the recovery from SCI.

## Author Contributions

Sioui Maldonado Bouchard wrote the manuscript, conducted the literature review, and proposed the hypothesis put forth. Michelle A. Hook provided extensive feedback and proof-reading, and contributed to the final form of the manuscript.

## Conflict of Interest Statement

The authors declare that the research was conducted in the absence of any commercial or financial relationships that could be construed as a potential conflict of interest.
